# Structural basis for varying drug resistance of SARS-CoV-2 M^pro^ E166 variants

**DOI:** 10.1128/mbio.02624-24

**Published:** 2025-06-02

**Authors:** Morgan A. Esler, Ke Shi, Joseph A. Rollie, Renee Delgado, Jyoti Vishwakarma, Agnieszka Dabrowska, Janani Prahlad, Seyed Arad Moghadasi, Reuben S. Harris, Hideki Aihara

**Affiliations:** 1 Department of Biochemistry, Molecular Biology and Biophysics, University of Minnesota 5635, Minneapolis, Minnesota, USA; 2 Institute for Molecular Virology, University of Minnesota, Minneapolis, Minnesota, USA; 3 Department of Biochemistry and Structural Biology, University of Texas Health San Antonio 542198, San Antonio, Texas, USA; 4 Howard Hughes Medical Institute, University of Texas Health San Antonio 14742, San Antonio, Texas, USA; Icahn School of Medicine at Mount Sinai, New York, New York, USA

**Keywords:** SARS-CoV-2, viral protease, antiviral drug, drug resistance mechanisms, crystal structure, Nirmatrelvir, Ensitrelvir, Bofutrelvir

## Abstract

Severe acute respiratory syndrome coronavirus 2 (SARS-CoV-2) main protease (M^pro^) has an essential role in the virus lifecycle and, accordingly, it is a target for antiviral drugs. Multiple studies have identified an M^pro^ mutation (E166V) that confers strong resistance to clinically relevant inhibitors, including nirmatrelvir, but the underlying mechanism is not fully understood. Here, we report on crystal structures of SARS-CoV-2 M^pro^ E166V in complex with nirmatrelvir, ensitrelvir, and bofutrelvir. The structures suggest that resistance is caused in part by the loss of a direct hydrogen bond and also, especially for nirmatrelvir, by a steric clash with the substituted valine residue. In comparison, the binding of bofutrelvir shows greater flexibility, which may help alleviate this steric effect and allow bofutrelvir to fit the mutant active site despite the loss of a direct polar contact. Thermal stability analyses also corroborate E166V most severely affecting the binding of nirmatrelvir and, to lesser and different extents, ensitrelvir and bofutrelvir. We further show that E166I causes even more severe nirmatrelvir resistance, whereas E166A and E166L have much milder effects. These studies shed light on the molecular mechanisms of a key M^pro^ drug resistance mutation and may help inform the design of next-generation inhibitors.

**IMPORTANCE:**

Using a combination of high-resolution X-ray crystallographic and biochemical analyses, we reveal the molecular mechanisms by which a mutation in the severe acute respiratory syndrome coronavirus 2 main protease (M^pro^) confers strong resistance against clinically relevant antiviral drugs that inhibit M^pro^ activity. The results presented here may help inform the design of next-generation inhibitors to combat the problem of therapy resistance.

## INTRODUCTION

The main/3C-like protease (M^pro^/3CL^pro^) of severe acute respiratory syndrome coronavirus 2 (SARS-CoV-2) is responsible for making 11 out of 14 cleavage events for liberating and activating the viral non-structural proteins essential for the virus replication, and accordingly, it is an important target for antiviral drugs (1–4). Clinically relevant SARS-CoV-2 M^pro^ inhibitors, including nirmatrelvir, the active component of the Food and Drug Administration-approved drug Paxlovid, and ensitrelvir, which has been approved in Japan for the treatment of coronavirus disease 2019, target the cysteine protease active site of M^pro^ to competitively block its catalytic activity (5, 6). M^pro^ mutations that confer resistance to these drugs have been identified through serial passaging of the virus in cell cultures under selective pressure, and some of such mutations have been observed in naturally circulating strains (7–10). As SARS-CoV-2 continues to evolve, there is a potential for M^pro^ inhibitor-resistant viruses to spread in the future (11). Understanding the molecular mechanisms of drug resistance is important for developing next-generation M^pro^ inhibitors to combat the problem of therapy resistance.

Among the drug resistance mutations in M^pro^ identified so far, a replacement of Glu166 by valine (E166V) elicits the strongest resistance to nirmatrelvir with a 100-fold increase in the antiviral EC_50_ relative to the wild-type virus (8), a 220-fold increase in biochemical IC_50_ in a peptide cleavage assay relative to the wild-type enzyme (12), and >300-fold increase of EC_50_ in a cell-based gain-of-signal relative to a wild-type M^pro^ construct (13, 14). The E166V mutation also confers strong resistance to ensitrelvir, although the fold increase in IC_50_ is smaller: 23-fold in the antiviral activity (8), ninefold in the biochemical assay (12), and 78-fold in the cell-based assay (13) relative to wild type. The Glu166 side chain interacts with the pyrrolidone moiety of nirmatrelvir and bofutrelvir (FB2001) and the triazole moiety of ensitrelvir, which mimic the side chain of the P1 Gln residue of the substrate peptide (5, 15, 16). Glu166 is also hydrogen-bonded with the N-terminal Ser1 of the dimer partner to stabilize the M^pro^ homodimer (17) and intramolecularly with His172 to stabilize its active site. Reflecting the importance of Glu166, the E166V mutation leads to a severe loss of enzymatic activity and viral replicative fitness (8, 10, 12). However, the activity of M^pro^ E166V can be restored by compensatory mutations, such as L50F and T21I, to levels that enable virus replication, and SARS-CoV-2 resistance to nirmatrelvir was shown to readily arise via multiple pathways in cell culture studies, suggesting the possibility for therapy-resistant viruses to spread (8–10). Curiously, a replacement of Glu166 by alanine (E166A), which would cause the same loss of polar contacts as E166V, confers much less resistance against nirmatrelvir (21-fold relative to wild type in the cell-based assay), whereas E166V and E166A confer similar levels of resistance against ensitrelvir and bofutrelvir (13). The molecular mechanisms underlying these complex resistance profiles are not fully understood. Here, we report on crystallographic data and biochemical analyses that help explain the drug resistance mechanisms of E166V and other related amino acid substitutions in SARS-CoV-2 M^pro^. Our studies show that E166V and E166I cause strong nirmatrelvir resistance, while E166A and E166L have a much milder effect, suggesting that the steric clash of a conformationally restrained side chain with the drug is primarily responsible for the resistance.

## RESULTS

### Structure determination

Initial attempts to crystallize SARS-CoV-2 M^pro^ with the single E166V mutation were not successful possibly due to compromised protein solubility. We reasoned that suppressor mutations, such as T21I and L50F, which have been shown to rescue the severe fitness defect of the virus carrying the M^pro^ E166V mutation (8–10), may also mitigate biochemical defects. We, therefore, tested multiple single, double, and triple mutants in combination with E166V. Of the several proteins screened, M^pro^ with four amino acid substitutions, namely, T21I, L50F, S144A, and E166V (hereafter referred to as QM for quadruple mutant), yielded high-quality crystals, allowing for structure determination by itself and in complex with nirmatrelvir, ensitrelvir, and bofutrelvir at resolutions ranging from 1.71 to 2.33 Å. We also determined the structures of M^pro^ E166V/L50F bound to ensitrelvir at 1.65 Å, M^pro^ E166V/T21I/L50F in complex with bofutrelvir at 2.09 Å, and wild-type M^pro^ in complex with bofutrelvir in two new crystal forms at 1.68 and 1.91 Å resolution. A summary of these crystal structures and data collection and model refinement statistics is shown in Table 1.

**TABLE 1:** X-ray data collection and refinement statistics^*a*^

	QM-apo (9CJO)	QM-nirmatrelvir (9CJP)	QM-ensitrelvir (9CJQ)	QM-bofutrelvir (9CJT)
Data collection				
Space group	C2	P2_1_	P2_1_	C2
Unit cell dimensions				
a, b, c (Å)	113.21, 53.67, 45.20	48.10, 105.67, 53.10	45.02, 106.99, 116.52	113.63, 53.89, 45.09
α, β, γ (º)	90, 101.55, 90	90, 103.62, 90	90, 99.34, 90	90, 102.20, 90
Resolution (Å)	48.2–2.33 (2.57–2.33)	52.8–1.70 (1.93–1.70)	78.33–2.24 (2.46–2.24)	34.2–1.92 (2.13–1.92)
Rsym or Rmerge	0.153 (0.630)	0.071 (0.470)	0.116 (0.787)	0.054 (0.600)
I/σI	4.6 (1.5)	6.1 (1.9)	5.8 (1.6)	7.2 (1.5)
Completeness (%)	82.8 (43.8)^*b*^	91.5 (66.6)^*c*^	90.5 (55.8)	85.5 (38.9)^*d*^
Redundancy	3.5 (3.2)	3.0 (2.7)	3.8 (3.3)	3.2 (3.2)
CC1/2	0.989 (0.597)	0.996 (0.785)	0.997 (0.663)	0.998 (0.702)
Refinement				
Resolution (Å)	44.3–2.33 (2.41–2.33)	46.7–1.73 (1.79–1.73)	78.33–2.25 (2.33–2.25)	31.21–1.92 (1.99–1.92)
No. of reflections	5,375 (37)	32,789 (204)	34,332 (196)	13,816 (88)
Rwork/Rfree	0.218/0.267	0.180/0.226	0.232/0.258	0.215/0.250
No. of atoms	2,336	4,846	9,697	2,434
Protein	2,329	4,667	9,374	2,377
Ligand/ion	0	86	152	33
Water	7	93	171	57
B-factor	49.46	43.48	45.6	54.83
Protein	49.49	43.64	45.89	54.88
Ligand/ion	n.a.	39.99	36.6	57.83
Water	41.05	38.35	37.53	52.71
Ramachandran plot				
Favored (%)	90.3	97.49	95.67	96
Allowed (%)	9.03	2.35	3.92	2.01
Outliers (%)	0.67	0.17	0.42	1
R.m.s. deviations				
Bond lengths (Å)	0.002	0.004	0.003	0.003
Bond angles (º)	0.43	0.73	0.73	0.61

	Wild type-bofutrelvir (9CJU)	Wild type-bofutrelvir (9CJV)	E166V/T21I/L50F-bofutrelvir (9CJS)	E166V/L50F-ensitrelvir (9CJR)
Data collection				
Space group	P2_1_2_1_2_1_	C2	C2	P2_1_
Unit cell dimensions				
a, b, c (Å)	67.85, 99.82, 104.11	54.44, 99.40, 57.08	113.18, 53.49, 44.935	54.44, 99.40, 57.08
α, β, γ (º)	90, 90, 90	90, 105.76, 90	90, 102.39, 90	90, 105.76, 90
Resolution (Å)	33.9–1.68 (1.86–1.68)	34.23–1.91 (2.05–1.91)	48.1–2.09 (2.32–2.09)	33.62–1.65 (1.84–1.65)
Rsym or Rmerge	0.129 (1.116)	0.041 (0.47)	0.208 (1.320)	0.067 (0.786)
I/σI	8.4 (1.0)	10.2 (1.5)	4.9 (1.4)	5.8 (1.6)
Completeness (%)	95.1 (65.5)^*e*^	85.3 (54.0)^*f*^	88.8 (45.4)^*g*^	88.3 (69.8)^*h*^
Redundancy	6.7 (6.1)	3.1 (3.4)	3.6 (3.6)	3.8 (4.1)
CC1/2	0.995 (0.587)	0.999 (0.668)	0.952 (0.307)	0.997 (0.595)
Refinement				
Resolution (Å)	33.9–1.68 (1.71–1.68)	34.23–1.91 (2.06–1.91)	48.15–2.09 (2.17–2.09)	33.1–1.65 (1.71–1.65)
No. of reflections	59,615 (2828)	12,715 (638)	9,804 (44)	39,566 (293)
Rwork/Rfree	0.175/0.210	0.218/0.246	0.215/0.272	0.177/0.216
No. of atoms	5,293	2,449	2,410	5,054
Protein	4,740	2,346	2,329	4,720
Ligand/ion	104	41	33	112
Water	449	62	48	256
B-factor	32.71	43.72	46.07	34.44
Protein	32.05	43.81	46.16	34.21
Ligand/ion	45.69	60.6	43.09	32.89
Water	38.7	38.12	41.52	39.21
Ramachandran plot				
Favored (%)	97.67	94.02	93.31	97.99
Allowed (%)	2.16	5.32	6.35	2.01
Outliers (%)	0.17	0.66	0.33	0
R.m.s. deviations				
Bond lengths (Å)	0.009	0.008	0.003	0.002
Bond angles (º)	1.018	1.311	0.6	0.55

^*a*^ Statistics for the highest-resolution shell are shown in parentheses.

^*b*^ a* 3.5 Å, b* 2.4 Å, −0.446a* + 0.895c* 2.2 Å.

^*c*^ 0.987a*–0.163c* 1.70 Å, b* 2.00 Å, −0.080a* + 0.997c* 2.29 Å.

^*d*^ a*–0.001c* 2.37 Å, b* 2.02 Å, −0.468a*+0.884c* 1.88 Å.

^*e*^ a* 1.68Å, b* 1.95Å, c* 1.88Å.

^*f*^ 0.964a*–0.264c* 2.63Å, b* 1.94Å, 0.771a* + 0.637c* 1.82Å.

^*g*^ 0.999a*–0.048c* 2.89 Å, b* 2.20 Å, −0.238a* + 0.971c* 2.04 Å.

^*h*^ 0.655a*–0.756c* 2.33 Å, b* 1.87 Å, 0.748a* + 0.663c* 1.62 Å.

### Activities and drug resistance of M^pro^ mutants

**Fig 1:**
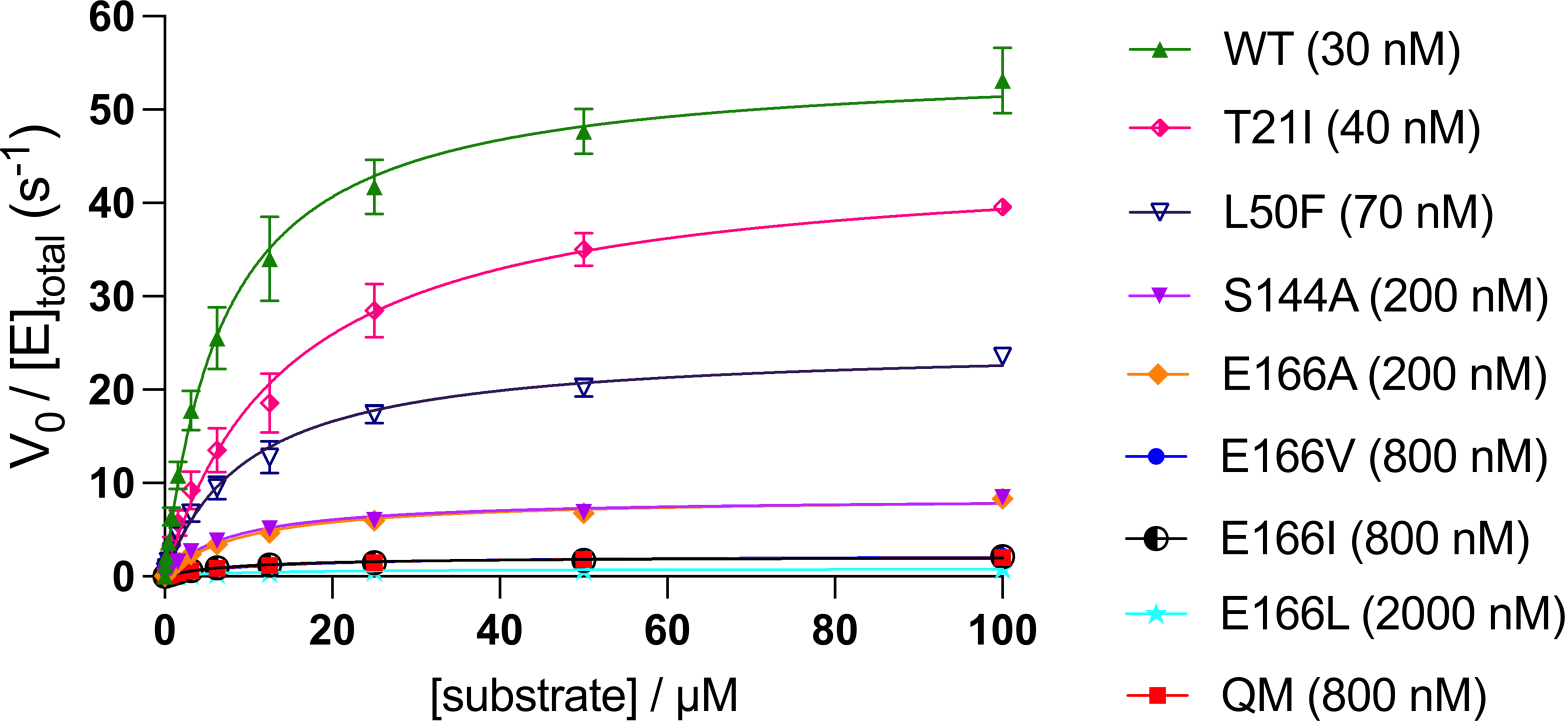
Biochemical activities of M^pro^ variants. Michaelis-Menten plot for wild-type M^pro^ and variants. The enzyme concentration used in the substrate titration experiment for each protein is shown in the key. The plotted values are the average and standard deviation of four experiments (two technical replicates and two biological replicates). A separate graph for E166A, E166V, E166I, E166L, and QM is shown in Fig. S1, and a summary of steady-state kinetic parameters obtained from these data is in Table 2. WT denotes wild type.

Steady-state kinetics of M^pro^ QM and the variants carrying individual amino acid substitutions (T21I, L50F, S144A, or E166V) were examined to evaluate the functional consequence of the mutations. In addition, we also tested M^pro^ E166A, E166L, and E166I, which have the substitution of a similar aliphatic side chain as E166V for Glu166. In a biochemical assay using an internally quenched fluorogenic substrate (Dabcyl-KTSAVLQSGFRKME-Edans), we determined the K_m_ of wild-type M^pro^ to be 7.1 µM, whereas T21I showed approximately twofold higher K_m_ of 15 µM, indicating slightly compromised substrate binding. The K_m_ values of all other mutant variants, including E166V and QM, were within twofold of that of wild-type M^pro^. By contrast, the catalytic constant of the mutants exhibited a much greater variation. In particular, E166V, E166I, and QM showed ~25-fold reduced *k*_cat_ compared to wild type, and E166L showed even poorer activity with ~65-fold lower *k*_cat_ than wild type (Fig. 1 and Table 2; Fig. S1). These results confirm that substitution of a longer aliphatic side chain for Glu166 severely compromises the catalytic activity of M^pro^ and indicate that key mutants, including M^pro^ QM and E166V, have similar enzymatic activities.

**TABLE 2:** Steady-state kinetic parameters for M^pro^ wild type and derivatives from data in Fig. 1

Enzyme (protein concentration used)	K_m_ (μM) (95% CI)	*k*_cat_ (s^−1^) (95% CI)
Wild type (30 nM)	7.1 (6.4 to 7.8)	55 (54 to 57)
T21I (40 nM)	15 (13 to 17)	45 (43 to 47)
L50F (70 nM)	9.9 (8.7 to 11)	25 (24 to 26)
S144A (200 nM)	7.7 (6.8 to 8.8)	8.4 (8.1 to 8.8)
E166A (200 nM)	9.4 (8.1 to 11)	8.6 (8.2 to 9.0)
E166V (800 nM)	12 (10. to 13)	2.3 (2.2 to 2.4)
E166I (800 nM)	8.3 (7.3 to 9.4)	2.1 (2.0 to 2.2)
E166L (2,000 nM)	10. (9.2 to 12)	0.85 (0.82 to 0.88)
QM (T21I/L50F/S144A/E166V) (800 nM)	9.3 (8.3 to 11)	2.2 (2.1 to 2.2)

**Fig 2:**
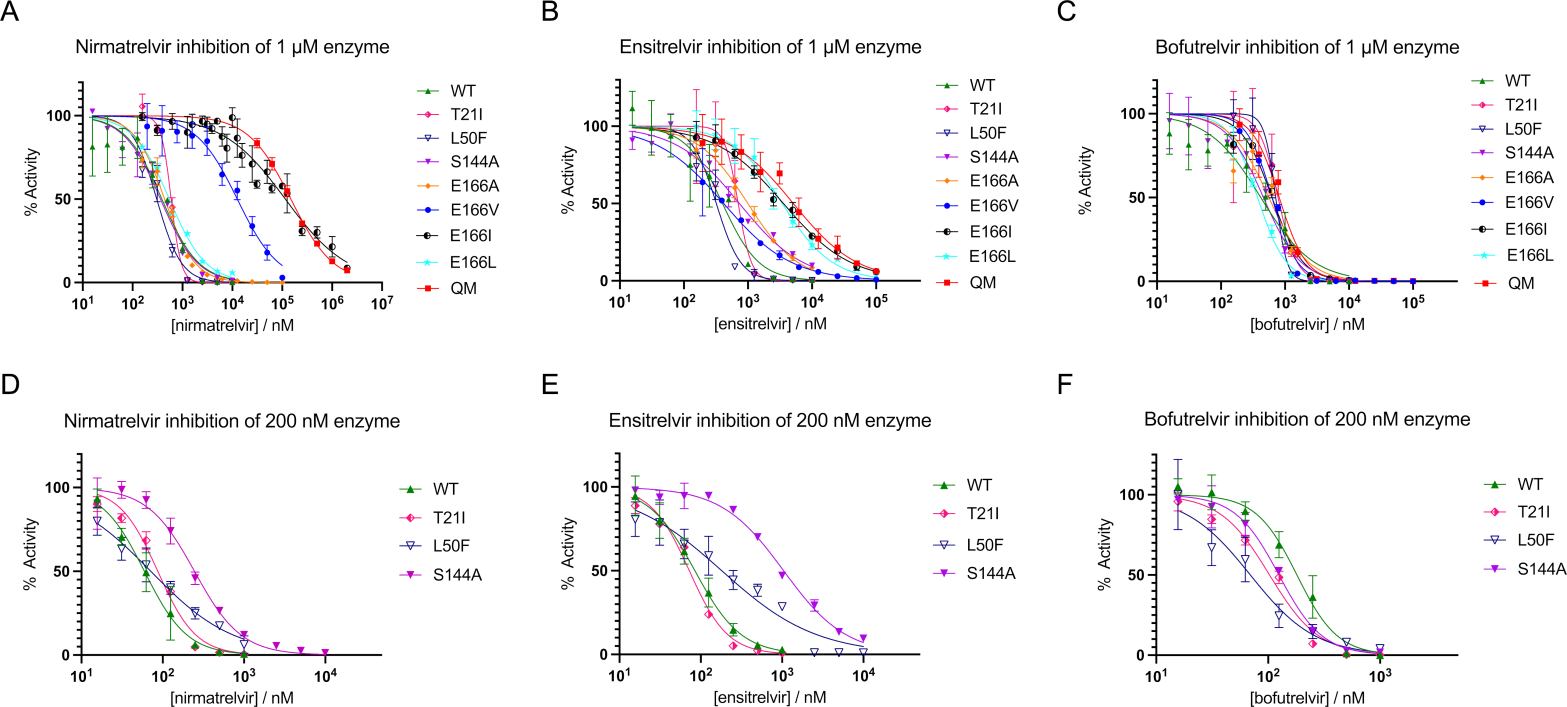
Inhibition of M^pro^ wild type and variants by nirmatrelvir, ensitrelvir, and bofutrelvir. (**A–C**) Dose-response titration experiment with 1.0 µM enzyme. (**D–F**) Dose-response titration experiment with 200 nM enzyme. The average values of at least three replicates with standard deviation are plotted. WT denotes wild type. The IC50 values are summarized in Tables 3 and 4.

We then examined the inhibition of wild-type and all M^pro^ variants at 1.0 µM enzyme concentration by nirmatrelvir, ensitrelvir, and bofutrelvir (Fig. 2A through C; Table 3) and in parallel at 200 nM enzyme concentration for those with less compromised activity (wild type, T21I, L50F, and S144A; Fig. 2D through F; Table 4). The use of the high enzyme concentration (1.0 µM) was necessary for evaluating the drug resistance of E166A/V/L/I that showed very low basal activity, whereas the data with 200 nM enzyme were more useful for making comparisons between more active and less drug-resistant variants. The biochemical activity of wild-type M^pro^ at either concentration was strongly inhibited by all three drugs. The IC_50_ values were 60 nM for nirmatrelvir, 85 nM for ensitrelvir, and 180 nM for bofutrelvir against 200 nM enzyme (Fig. 2D through F; Table 4) and 390 nM for nirmatrelvir, 400 nM for ensitrelvir, and 420 nM for bofutrelvir against 1.0 µM enzyme following the “tight-binding inhibition” regime (18) (Fig. 2A through C; Table 3). As expected, the E166V mutation caused strong resistance against nirmatrelvir with an IC_50_ of 13 µM but with small or no effect on ensitrelvir and bofutrelvir inhibition when tested against 1.0 µM enzyme (Fig. 2A through C). The QM variant showed even greater resistance against nirmatrelvir (IC_50_ = 150 µM) and also strong resistance against ensitrelvir (IC_50_ = 5.1 µM) but not against bofutrelvir (IC_50_ = 810 nM; Fig. 2A through C). A similarly high level of nirmatrelvir resistance was observed for E166I, with an IC_50_ of 120 µM, whereas E166A and E166L did not show obvious resistance against nirmatrelvir (Fig. 2A). On the other hand, E166L and E166I conferred stronger ensitrelvir resistance than E166A or E166V, similar to QM (Fig. 2B). Aside from the E166 variant series, T21I showed notably steeper (greater negative) Hill slopes against all three drugs tested against 1.0 µM enzymes, which may indicate enhanced cooperativity (Fig. 2A through C). The inhibition profiles for 200 nM enzymes highlighted that S144A confers strong ensitrelvir resistance and weaker but significant nirmatrelvir resistance while having no impact on bofutrelvir (Fig. 2D through F). The data with 200 nM enzyme also show that L50F causes modest ensitrelvir resistance. These observations explain why QM shows greater resistance against nirmatrelvir and ensitrelvir than E166V alone. We did not observe strong bofutrelvir resistance from any of the variants at either of the enzyme concentrations (Fig. 2C and F). The IC_50_ values are summarized in Tables 3 and 4. Of note, varying the duration of incubation for wild-type or E166V M^pro^ with the reversible, covalent inhibitors nirmatrelvir and bofutrelvir did not have a strong impact on their sensitivity to these drugs (Fig. S2). Thus, the resistance observed for these drugs is not caused by the slower kinetics of the covalent adduct formation.

**TABLE 3:** IC_50_ values for the three drugs against 1.0 µM M^pro^ wild type and variants^*a*^

	Nirmatrelvir	Ensitrelvir	Bofutrelvir
Wild type (1.0 µM)	390 nM (300 to 490)	400 nM (320 to 500)	420 nM (320 to 540)
T21I	590 nM (570 to 620 nM)	700 nM (590 to 830)	780 nM (610 to 970)
L50F	310 nM (270 to 350 nM)	320 nM (280 to 360)	710 nM (ND to 780)
S144A	360 nM (320 to 410 nM)	740 nM (620 to 870)	460 nM (380 to 550)
E166A	440 nM (410 to 470 nM)	1.0 µM (900 nM to 1.1 µM)	560 nM (440 to 700 nM)
E166V	13 µM (10. to 15 µM)	420 nM (340 to 500 nM)	630 nM (590 to 660 nM)
E166I	120 µM (98 to 140 µM)	3.6 µM (3.1 to 4.1 µM)	630 nM (570 to 690 nM)
E166L	550 nM (520 to 580 nM)	3.4 µM (3.0 to 4.0 µM)	370 nM (350 to 400 nM)
QM (T21I/L50F/S144A/E166V)	150 µM (140 to 160 µM)	5.1 µM (4.0 to 6.5 µM)	810 nM (740 to 900 nM)

^*a*^ The IC_50_ values are based on data plotted in Fig. 2A through C, which summarize results from at least three replicates. The 95% confidence intervals estimated by GraphPad Prism are shown in parentheses. ND, not determined.

**TABLE 4:** IC_50_ values for the three drugs against 200 nM M^pro^ wild type and variants^*a*^

	Nirmatrelvir	Ensitrelvir	Bofutrelvir
Wild type (200 nM)	60. nM (53 to 67)	85 nM (76 to 95)	180 nM (160 to 200)
T21I	87 nM (79 to 95)	72 nM (67 to 78)	100 nM (93 to 110)
L50F	71 nM (61 to 83)	180 nM (140 to 230)	69 nM (56 to 86)
S144A	250 nM (220 to 280)	1.0 µM (960 nM to 1.1 µM)	130 nM (120 to 140)

^*a*^ The IC_50_ values are based on data plotted in Fig. 2D through F, which summarize results from at least three replicates. The 95% confidence intervals estimated by GraphPad Prism are shown in parentheses.

### Overall structure of M^pro^ mutants

**Fig 3:**
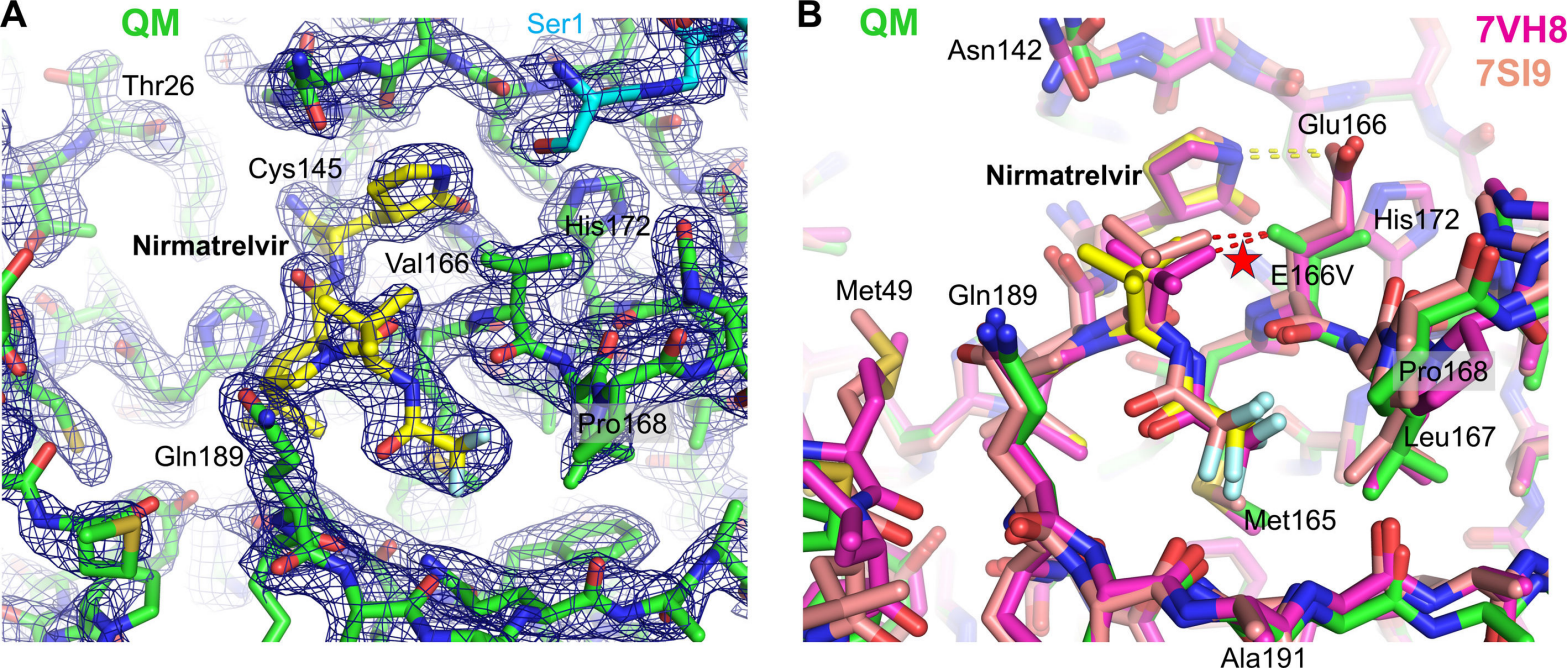
Structural basis of nirmatrelvir resistance. (A) M^pro^ QM (E166V/T21I/L50F/S144A) structure in complex with nirmatrelvir at 1.70 Å resolution with the 2Fo-Fc electron density contoured at 1.0σ in blue mesh. The two M^pro^ molecules from a homodimer are colored green and cyan, whereas nirmatrelvir is shown in yellow (for carbon atoms). Non-carbon atoms are colored according to the atom type. (**B**) Superposition of the M^pro^ QM-nirmatrelvir structure (colored as in **A**) with the previously published wild-type M^pro^-nirmatrelvir structures PDB 7VH8 (20) and 7SI9 (21) in magenta and tan, respectively. The red dashed lines and star indicate that there would be a clash between the Cγ^1^ atom of the substituted Val166 side chain and the *tert*-butyl group of nirmatrelvir at the original position. The yellow dashed lines indicate a hydrogen bond between Glu166 and the pyrrolidone ring of nirmatrelvir.

**Fig 4:**
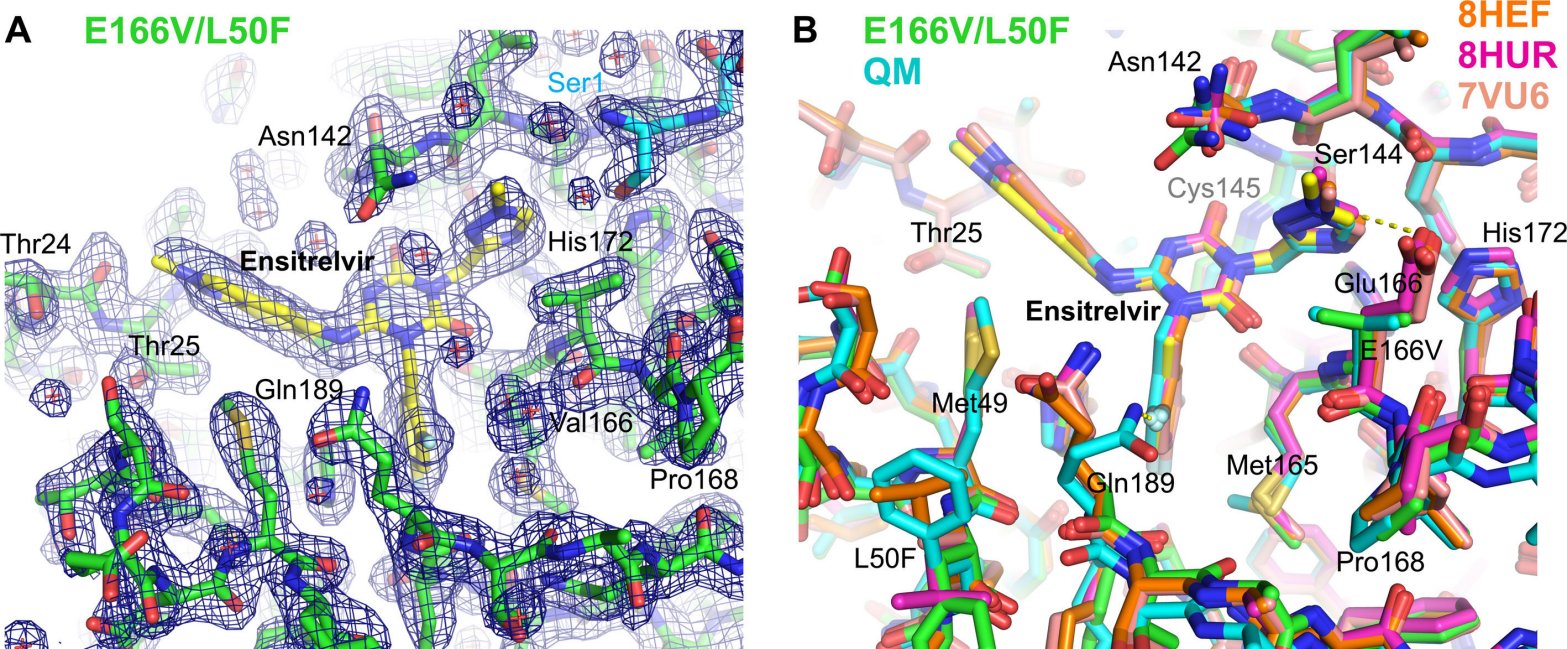
Structural basis of ensitrelvir resistance. (A) M^pro^ E166V/L50F structure in complex with ensitrelvir at 1.65 Å resolution with the 2Fo-Fc electron density contoured at 1.0σ in blue mesh. The two M^pro^ molecules from a homodimer are colored green and cyan, whereas ensitrelvir is shown in yellow (for carbon atoms). Non-carbon atoms are colored according to the atom type. (**B**) Superposition of the M^pro^ E166V/L50F-ensitrelvir structure (colored as in **A**) and the M^pro^ QM-ensitrelvir structure at 2.24 Å resolution (cyan) with the previously published wild-type M^pro^-ensitrelvir structures (PDB 8HEF [22], 8HUR [16], and 7VU6 [6] in orange, magenta, and tan, respectively). The yellow dashed lines indicate an interaction between Glu166 and the triazole ring and a short-distance hydrogen bond between Gln189 and a fluorine atom from ensitrelvir.

The structures of M^pro^ QM, E166V/T21I/L50F, and E166V/L50F all show a symmetric homo-dimeric assembly very similar to that of the wild-type enzyme. The structures can be superimposed with most previously reported wild-type M^pro^ structures (e.g., 7BQY) (19) with the root-mean-square-deviation for the backbone atoms smaller than 0.5 Å, suggesting that the four amino acid substitutions of QM do not cause major structural changes. The substituted amino acids and the surrounding residues are well resolved in the electron density map, except for the region including Phe50 (L50F) in the nirmatrelvir-bound M^pro^ QM structure that has weak electron density and is presumably disordered. The electron density map shows that the substituted Val166 in the active site takes the rare gauche^+^ side chain rotamer (*χ*_1_ angle = +60°)(Fig. 3A, 4A, and 5A). This is likely because the more common *trans* (*χ*_1_ angle = 180°) and gauche^–^ (*χ*_1_ angle = –60°) conformations would position one of the two Cγ atoms ~2.6 Å from the His172 side chain and cause a severe steric clash (Fig. S3). This valine side-chain conformation affects the binding of nirmatrelvir and bofutrelvir, as discussed below. As expected for the substitution of a non-polar valine residue for glutamic acid, the E166V mutation abolishes the intramolecular and intermolecular hydrogen bonds with the side chains of His172 and Ser1, respectively, which stabilize the S1 pocket of the M^pro^ active site. Ser1 maintains its proximity to the active site within M^pro^ dimers through alternative interactions as discussed below.

**Fig 5:**
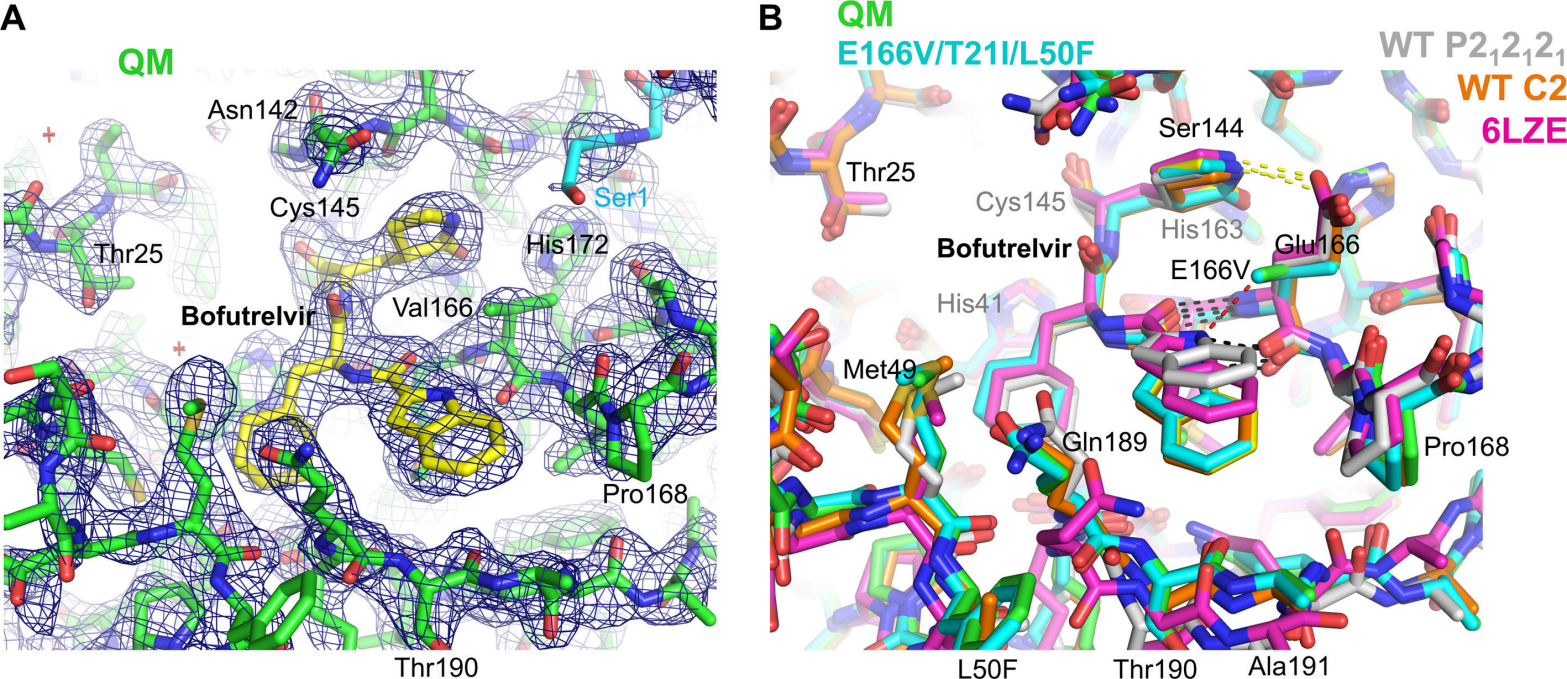
Structural basis of bofutrelvir resistance. (A) M^pro^ QM structure in complex with bofutrelvir at 1.92 Å resolution with the 2Fo-Fc electron density contoured at 1.0σ in blue mesh. The two M^pro^ molecules from a homodimer are colored green and cyan, whereas ensitrelvir is shown in yellow (for carbon atoms). Non-carbon atoms are colored according to the atom type. (**B**) Superposition of the M^pro^ QM-bofutrelvir structure (colored as in **A)**, M^pro^ E166V/T21I/L50F-bofutrelvir structure at 2.09 Å resolution (cyan), wild-type M^pro^-bofutrelvir structure at 1.91 Å resolution in the C2 space group determined in this study (orange), a previously published wild-type M^pro^-bofutrelvir structure (PDB 6LZE) (15) in magenta, and wild-type M^pro^-bofutrelvir structure at 1.68 Å resolution in the P2_1_2_1_2_1_ space group determined in this study (gray). The yellow dashed lines indicate a hydrogen bond between Glu166 and the pyrrolidine ring of bofutrelvir. The red dashed line highlights that Val166 and a nitrogen atom of bofutrelvir in the “indole-up” conformation would be unfavorably close (3.0 Å). The black dashed lines indicate bidentate hydrogen bonds between bofutrelvir and the Glu/Val166 main chain persistently formed despite the large variability in the positioning of the indole moiety. WT denotes wild type.

### Mechanism of nirmatrelvir resistance

The structure of M^pro^ QM in complex with nirmatrelvir shows the inhibitor covalently bonded to Cys145 in the active site, as observed in the wild type, as well as various other mutant M^pro^ structures with nirmatrelvir (20, 21) (Fig. 3A). A superposition with the wild-type M^pro^ structure bound to nirmatrelvir shows that the positioning of the pyrrolidone ring occupying the S1 pocket is minimally affected, although a hydrogen bond between Glu166 and the pyrrolidone ring is lost due to the E166V mutation (Fig. 3B). The side chain of Ser1 in turn forms a hydrogen bond network to bridge between His172 and the pyrrolidone moiety. S144A replaces Ser144, which interacts weakly with the pyrrolidone ring in wild-type M^pro^-nirmatrelvir structures (4.1 Å between Ser Oγ and the carbonyl oxygen from pyrrolidone; PDB IDs: 7VH8 and 7SI9) and is also hydrogen-bonded to the main chain of Leu141 lining the S1 pocket. The observed nirmatrelvir resistance of S144A could reflect perturbation to either of these interactions.

The main chain atoms of Val166 are engaged in β-sheet-like hydrogen bonds with those of a 3-methyl-valine group of nirmatrelvir that mimics the P3 residue of the substrate, as observed for Glu166 in the previous structures. However, the trimethyl (*tert*-butyl) group of the 3-methyl-valine side chain shows a significant deviation in its positioning compared to the previous M^pro^-nirmatrelvir structures, shifted by ~0.8 Å away from the Val166 Cγ^1^ methyl group to mitigate a steric clash (yellow/green in Fig. 3B). Without the shift, a methyl group carbon atom from nirmatrelvir would be within 3 Å (e.g., 2.7 Å based on superposition to 7VH8 [20]) from the Val166 side chain Cγ^1^ atom (the clash indicated by red dashed lines and a star in Fig. 3B), which is constrained to take the gauche^+^ side chain rotamer due to the presence of two Cγ atoms in valine (Fig. S3). This severe steric clash necessitates nirmatrelvir to bind in a strained conformation to the M^pro^ active site with the E166V mutation, which likely contributes to the strong resistance along with the loss of the hydrogen bond. The observation explains why E166I also confers strong nirmatrelvir resistance, whereas E166L and E166A do not. An isoleucine side chain with two Cγ atoms would be constrained similarly to the valine in E166V and suffer from a steric clash with nirmatrelvir, whereas leucine with only one Cγ atom can take a side chain conformation similar to that of Glu166 in wild-type M^pro^ to avoid such clashes. An alanine (E166A) side chain without a Cγ atom would not have a steric clash with nirmatrelvir either.

### Ensitrelvir

The structures of M^pro^ QM and E166V/L50F in complex with ensitrelvir show that the positioning of the inhibitor in the active site is similar to that observed in the previous M^pro^-ensitrelvir structures (e.g., PDB 8HUR [16]) (Fig. 4A, yellow/green and cyan in Fig. 4B). The E166V mutation abolishes a direct polar contact between Glu166 and the edge of the triazole ring. However, in contrast to the case with nirmatrelvir, the substituted valine residue does not cause a steric clash with ensitrelvir; the Val166 side chain is positioned 3.7 Å from the triazole ring of ensitrelvir for a favorable van der Waals contact, which may explain why E166V confers less drug resistance than E166A specifically against ensitrelvir (Fig. 2B and Table 3). The S144A mutation in the QM also causes a loss of direct polar interaction with the triazole moiety of ensitrelvir (3.5 Å between Ser Oγ and a nitrogen atom from triazole in our M^pro^ E166V/L50F-ensitrelvir structure), although it does not significantly affect the positioning of the inhibitor. Ser1 from the dimer partner takes varying conformations, where either its side chain or the N-terminal amino group interacts with the His172 side chain. Overall, the structures suggest that the M^pro^ E166V and S144A mutations affect the binding of ensitrelvir via loss of direct polar contacts. Notably, in two of the four molecules in the asymmetric unit of the M^pro^ QM structure bound to ensitrelvir, the Gln189 side chain takes a bent conformation and makes a short-distance (2.1–2.2 Å) fluorine-amine hydrogen bond with the trifluorobenzyl moiety of ensitrelvir (cyan in Fig. 4B). This unique conformer may result from the neighboring L50F mutation and could be strengthening the binding to ensitrelvir. However, it was not observed in the M^pro^ E166V/L50F structure bound to ensitrelvir while being also observed in wild-type M^pro^ bound to bofutrelvir (see below, PDB 6LZE (15)), and, thus, it might represent a conformation sampled by inhibitor-bound M^pro^, independently of the amino acid changes analyzed here.

### Bofutrelvir

The structures of M^pro^ QM and E166V/T21I/L50F bound to bofutrelvir (FB2001) at 1.92 and 2.09 Å resolutions, respectively, show that bofutrelvir covalently bonded to Cys145 in the active site (Fig. 5A, yellow/green and cyan in Fig. 5B). The two structures exhibit no significant difference, except for the replacement of the Ser144 side chain, which caused little structural perturbation. The E166V mutation abolishes a hydrogen bond from Glu166 to the pyrrolidone moiety of bofutrelvir, as expected for the replacement of Glu by non-polar Val, but the positioning of the pyrrolidine ring is minimally affected. On the contrary, the position of the indole ring of bofutrelvir occupying the S4 pocket shows a significant (>2 Å) deviation from that in the wild-type M^pro^ structure in complex with bofutrelvir reported previously (15) (PDB 6LZE; magenta in Fig. 5B)—the indole ring lies flat and is bound more deeply in the peptide-binding groove in our mutant structures. The Glu/Val166 main chain forms bidentate hydrogen bonds with the indole-carboxamide group of bofutrelvir in both conformations.

To investigate whether the difference in the positioning of the indole moiety was due to the amino acid substitutions, we also determined the wild-type M^pro^-bofutrelvir complex structures in two different space groups at 1.68 and 1.91 Å resolutions, respectively (Table 1). The former structure showed an “indole up” conformation similar to 6LZE but with the indole ring bound even more shallowly (gray in Fig. 5B), whereas the latter showed the same “indole down” conformation (orange in Fig. 5B) as M^pro^ QM and E166V/T21I/L50F. Thus, the variable positioning of the indole moiety was not caused by the E166V mutation but rather seems to reflect the flexibility of bofutrelvir binding. Regardless, the indole-down conformation appears to relieve a close contact between the Cγ^1^ methyl group of Val166 and the indole nitrogen atom. The carbon–nitrogen distance would be too short at 3.0 Å in the indole-up conformation, whereas it is 3.6 Å in the indole-down conformation for a more favorable van der Waals interaction. The M^pro^ loop centered on Thr190 concomitantly moves up toward bofutrelvir by ~1.5 Å in the mutant structures. These rearrangements allow bofutrelvir to form more intimate van der Waals contacts with the main chain atoms of Thr190 and Ala191 and the Gln189 side chain, making it fit better in the mutant M^pro^ active site. This may compensate for the loss of a hydrogen bond to the pyrrolidone group and minimize the reduction of affinity caused by the E166V mutation. In our 1.68 Å resolution structure (gray in Fig. 5B) and the previously reported structure (magenta in Fig. 5B, PDB 6LZE) of wild-type M^pro^-bofutrelvir complexes in the indole-up conformation, ethylene glycol and dimethylsulfoxide (DMSO) molecule, respectively, is bound underneath the indole group, filling a void between bofutrelvir and the protein. On the contrary, in our mutant (QM and E166V/T21I/L50F) and wild-type (1.91 Å) M^pro^-bofutrelvir structures, there is insufficient space for an additional ligand due to the shifts mentioned above and a reorientation of the terminal methyl group of the Met165 side chain. The flexibility represented by these structural variations may help alleviate the steric effect and allow bofutrelvir to fit the mutant active site despite the loss of a polar contact.

### Impact of E166V on drugs binding

**Fig 6:**
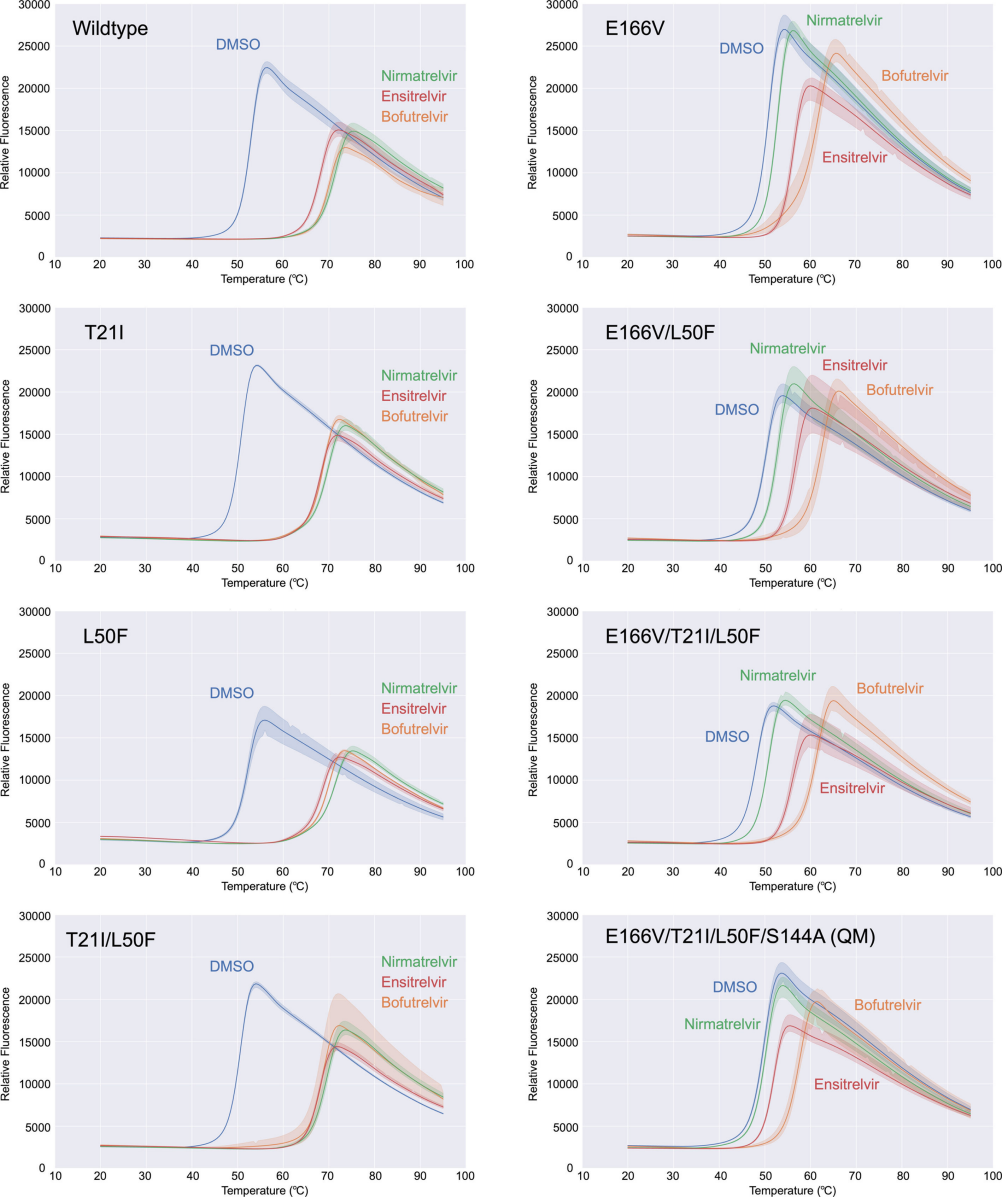
Thermal stability of M^pro^ variants and their interaction with nirmatrelvir, ensitrelvir, and bofutrelvir. Differential scanning fluorimetry melt curves for wild-type and various mutants of M^pro^ in the absence (DMSO control) and presence of three drugs. Average fluorescence intensities from triplicated experiments are plotted, with the ranges between the smallest and largest observed values indicated. The *x*-axis labels show the temperature in °C. The *y*-axis labels show fluorescence intensity in an arbitrary unit.

The thermal stability of various M^pro^ mutants and their physical interaction with the three different inhibitors were probed using differential scanning fluorimetry (DSF) (Fig. 6). Wild-type M^pro^ showed a monophasic melting profile, giving a melting temperature (*T*_m_) of 53.0°C. All mutants tested showed similar melting profiles but with slightly lower *T*_m_ compared to the wild type (Table 5): the E166V mutation elicited Δ*T*_m_ of –2.0°C. Similar destabilization was observed for T21I (Δ*T*_m_ = –2.0°C), while the effect of L50F was smaller (Δ*T*_m_ = –0.5°C). T21I/L50F and E166V/L50F both showed an additive effect of two mutations (*T*_m_ = 50.5°C, Δ*T*_m_ = –2.5°C). The triple mutant E166V/T21I/L50F showed the lowest *T*_m_ among all variants tested (*T*_m_ = 48.2°C, Δ*T*_m_ = –4.8°C). M^pro^ QM containing all these three mutations along with S144A showed a higher *T*_m_ of 50.0°C (Δ*T*_m_ = –3.0°C), indicating that S144A has a stabilizing effect.

**TABLE 5:** Melting temperature (T_m_ in °C) of M^pro^ variants in the absence or presence of inhibitors^*a*^

	DMSO	Nirmatrelvir	Ensitrelvir	Bofutrelvir
Wild type	53.0	71.5	68.2 ± 0.3	70.3 ± 0.3
T21I	51.0	70.0	68.0	69.0
L50F	52.5	71.5	68.8 ± 0.3	70.0
T21I/L50F	50.5	69.8 ± 0.3	68.0	68.8 ± 0.3
E166V	51.0	53.0	56.2 ± 0.3	61.8 ± 0.3
E166V/L50F	50.5	53.0	56.8 ± 0.3	62.7 ± 0.3
E166V/T21I/L50F	48.2 ± 0.3	51.0	56.0	61.5
E166V/T21I/L50F/S144A (QM)	50.0	50.5	52.0	57.7 ± 0.3

^*a*^ The average values (± standard deviation) from triplicated measurements are shown. T_m_ is the same between the three measurements when a standard deviation is not shown.

In comparison, the presence of a saturating concentration (100 µM) of nirmatrelvir, ensitrelvir, and bofutrelvir greatly stabilized wild-type M^pro^ and increased *T*_m_ to 71.5°C (Δ*T*_m_ = 18.5°C), 68.2°C (Δ*T*_m_ = 15.2°C), and 70.3°C (Δ*T*_m_ = 17.3°C), respectively, reflecting the high-affinity binding of these drugs (covalent for nirmatrelvir and bofutrelvir). In contrast, nirmatrelvir elicited Δ*T*_m_ of a mere 2.0°C for M^pro^ E166V, indicating a significant loss of affinity. The Δ*T*_m_ for M^pro^ E166V elicited by ensitrelvir and bofutrelvir was 5.2 and 10.8°C, respectively, suggesting that the E166V mutation has less impact on the binding of ensitrelvir and the least on bofutrelvir. The T21I and L50F mutations did not have a significant impact on drug binding, with M^pro^ T21I, L50F, and T21I/L50F all showing a similar trend of *T*_m_ shifts to wild-type M^pro^. M^pro^ QM showed the smallest Δ*T*_m_ among all variants for all three drugs: 0.5°C for nirmatrelvir, 1.5°C for ensitrelvir, and 7.7°C for bofutrelvir, consistent with its strongest drug resistance observed in the biochemical assays above for nirmatrelvir and ensitrelvir. These DSF results are consistent with the biochemical and structural observations described above and recapitulate the varying effects of E166V on different inhibitors.

## DISCUSSION

Our structural observations suggest that, even though the E166V mutation causes a loss of direct polar contact in the S1 pocket with nirmatrelvir, ensitrelvir, and bofutrelvir, the substituted valine residue also has distinct steric effects on the binding of these three inhibitors. Specifically, E166V compromises nirmatrelvir binding severely via a steric clash while supporting a weak van der Waals contact with ensitrelvir and potentially compensating for the loss of hydrogen bond for bofutrelvir binding through enhanced van der Waals contacts. The DSF data corroborate that the reduction of affinity caused by the E166V mutation is the greatest for nirmatrelvir, less for ensitrelvir, and the least for bofutrelvir. These results are fully consistent with our enzymatic activity and inhibition data, as well as reported resistance studies using a cell-based assay, which showed that the E166V mutation elicits >300-fold resistance to nirmatrelvir while having less (78-fold) effect on ensitrelvir and the least resistance (24-fold) on bofutrelvir (13). Our studies also explain why E166A causes only a 21-fold resistance to nirmatrelvir in a cell-based assay (13) and at least an order of magnitude smaller loss of susceptibility of SARS-CoV-2 replication to this drug (23). While valine and alanine substitutions both lead to the loss of a hydrogen bond to the pyrrolidone moiety, an alanine substitution does not have Cγ atoms and, therefore, would not cause steric clashes with nirmatrelvir. Similarly, a leucine substitution (E166L) has much less impact on nirmatrelvir binding than E166V or E166I because Leu does not have two Cγ atoms like Val or Ile, as discussed above. The even greater nirmatrelvir resistance of E166I compared to E166V could be due to the interaction of its additional methyl (CH_3_^δ1^) group with the surrounding residues (e.g., Gly170, His172, and Ser1) affecting the active site structure or dynamics to further compromise drug binding. It is notable that E166A/V/L/I showed distinct resistance profiles against nirmatrelvir vs. ensitrelvir. The stronger resistance of E166L/I against ensitrelvir than E166A/V may reflect an unfavorable interaction between the larger aliphatic side chains and the methyl-triazole moiety of ensitrelvir, a mechanism distinct from that for nirmatrelvir.

The DSF data showed that T21I and L50F, either individually or together, further de-stabilized (with negative Δ*T*_m_) M^pro^ E166V, which was partially rescued by S144A in the absence of a drug (Fig. 6 and Table 5). The observations suggest that the compensatory effects of the T21I and L50F mutations in rescuing the loss of viral fitness caused by the E166V mutation (8) are not due to protein stabilization. In contrast, recent studies showed that the activity loss of SARS-CoV-2 M^pro^ E166V is partially rescued by T21I and L50F, whereas S144A negatively impacts the activity (12). Thus, the very similar steady-state enzyme kinetic parameters we observed for E166V and QM (Fig. 1 and Table 2; Fig. S1) could result from the combined effects of these mutations exhibiting opposing effects in enzymatic activity and protein stability.

Bofutrelvir binding to the M^pro^ active site showed significant variability in the positioning of the indole moiety (Fig. 5). This observation highlights a flexibility that allows bofutrelvir to engage both the backbone amide nitrogen and carbonyl oxygen atoms for hydrogen bonding without clashing with the substituted Val166 side chain of the E166V mutant. The flexibility may also allow bofutrelvir to evade resistance caused by larger aliphatic side chain substitutions, E166I and E166L (Fig. 2C). This contrasts with nirmatrelvir, whose *tert*-butyl moiety (3-methyl-valine side chain) clashes with Val166 and suffers from severe resistance. Based on these observations, we speculate that a modification or removal of the *tert*-butyl group of nirmatrelvir would mitigate the drug resistance problem of M^pro^ variants containing the E166V or E166I. Recent studies have indeed demonstrated that changing a *tert*-butyl group at the position mimicking the P3 residue of the substrate to a smaller isopropyl group makes the newer-generation M^pro^ inhibitor, ML2006a4, less sensitive to the E166V-mediated resistance (24). An earlier study also showed that serial passaging of wild-type SARS-CoV-2 in cell culture in the presence of a first-generation M^pro^ inhibitor ALG-097161, which lacks a bulky moiety at the P3 position, selected a combination of mutations, including E166A rather than E166V (9). Our observations are consistent with these studies, as well as recent structural work on M^pro^ E166V and T21I/E166V in complex with nirmatrelvir (PDB IDs: 8H82 and 8H51) (12), which showed similar displacement of the drug caused by a steric clash with the valine side chain as that described here.

## MATERIALS AND METHODS

### Protein purification

Wild-type SARS-CoV-2 M^pro^ (USA-WA1, GenBank MT246667.1) and various mutant derivatives were expressed with an N-terminal 6xHis-Sumo fusion tag in *Escherichia coli* strain BL21(DE3) from codon-optimized expression plasmids. The expressed proteins in the soluble bacterial lysate were captured by a nickel affinity column and eluted with a linear concentration gradient of imidazole. Pooled fractions were treated with Sumo protease Ulp1 to remove the 6xHis-Sumo tag and further purified by Superdex 75 size-exclusion chromatography. Purified proteins in 20 mM Tris-HCl, pH 8.0, 150 mM NaCl, and 1 mM dithiothreitol (DTT) were concentrated to 12 mg mL^−1^ as determined by UV absorbance measured on a NanoDrop 8000 spectrophotometer and flash-frozen in liquid nitrogen for storage at −80°C.

### X-ray crystallography

Wild-type or mutant derivatives of M^pro^ at 12 mg mL^−1^ in the absence or the presence of 2.0 mM inhibitor and 2.5% DMSO were subjected to crystallization screening by sitting drop vapor diffusion method. Crystals were obtained with the following reservoir solutions: 0.1 M succinic acid pH 7.0, 15% (w/v) polyethylene glycol (PEG) 3350 for QM-APO, 0.1 M Tris-Bicine, pH 8.5, 0.09 M NPS, 12.5% (v/v) MPD, 12.5% w/v PEG1000, 12.5% w/v PEG3350 for QM-nirmatrelvir, 0.05 M HEPES sodium pH 7.0, 1% (w/v) Tryptone, 12% (w/v) PEG3350 for QM-ensitrelvir, 0.1 M MMT (DL-malic acid:MES:Tris base, 1:2:2) pH 6.0, 25% (w/v) PEG1500 for QM-bofutrelvir, 0.1 M Tris-Bicine, pH 8.5, 0.12 M ethylene glycol, 12.5% (v/v) MPD, 12.5% (w/v) PEG1000, 12.5% (w/v) PEG3350 for wild type-bofutrelvir (P2_1_2_1_2_1_ space group), 0.1 M Bis-Tris propane, pH 9.0, 25% (w/v) PEG1500, 0.1 M sodium chloride for wild type-bofutrelvir (C2 space group), 0.1 M MMT, pH 7.0, 25% (w/v) PEG1500 for E166V/T21I/L50F-bofutrelvir, 0.03 M sodium fluoride, 0.03 M sodium bromide, 0.03 M sodium iodide, 0.1 M HEPES-MOPS, 12.5% (v/v) MPD, 12.5% (w/v) PEG1000, and 12.5% (w/v) PEG3350 for E166V/L50F-ensitrelvir. Up to 25% ethylene glycol was used as a cryo-protectant. X-ray diffraction data were collected at the Northeastern Collaborative Access Team (NE-CAT) beamlines 24-ID-C and 24-ID-E of the Advanced Photon Source (Lemont, IL) or the AMX beamline of NSLS-II (Upton, NY) and processed using XDS (25) for integration, followed by three other programs from the CCP4 Suite (26): POINTLESS (27), AIMLESS (28), and TRUNCATE (https://doi.org/10.1107/S0567739478001114) for reduction, scaling, and structure factor calculation, respectively. Anisotropic diffraction analysis and truncation were done with STARANISO (https://staraniso.globalphasing.org/). The structures were determined by molecular replacement with PHASER (29) using our previously reported crystal structure of M^pro^ (PDB ID 7TGR [14]) as the search model. Iterative model building and refinement were conducted using COOT (30) and PHENIX (31). A summary of the crystallographic data statistics is shown in Table 1.

### Enzyme activity assay

The biochemical activity of M^pro^ was analyzed using a quenched fluorogenic peptide substrate DABCYL-KTSAVLQ↓SGFRKM-EDANS. M^pro^ cleavage of this peptide between Gln and Ser liberates fluorescence, which was quantified by excitation and emission at 350 and 490  nm, respectively, on a Tecan Spark 10M plate reader. The reactions were carried out in Corning 96-well half-area black flat bottom plates with 20 mM Tris-HCl, pH 8.0, 150 mM NaCl, 1 mM DTT, 1.0 mM EDTA, 0.05% Tween-20, and 0.1 mg mL^−1^ bovine serum albumin, in addition to the substrate and the enzyme. The measured fluorescence intensities were corrected for the inner filter effect based on the fluorescence intensity of EDANS mixed with various concentrations of the quenched peptide substrate (32) before being converted to the concentration of the cleaved substrate. Fluorescence readout data were converted to reaction velocity terms using the ICEKAT Enzyme Kinetics Software (33). The steady-state enzyme kinetics parameters were determined by fitting the data to the Michaelis-Menten model in GraphPad Prism. For inhibition studies, 200 nM or 1.0 µM M^pro^ was incubated with various concentrations of the inhibitors for 5 min at room temperature before being mixed with the peptide substrate at the final concentration of 30 µM to initiate the reaction. The initial velocities of the proteolytic reaction determined using ICEKAT were converted to % activity values by dividing them by the initial velocity of an uninhibited reaction. The IC_50_ values of inhibition for various enzyme-drug combinations were obtained by fitting the dose-response data via non-linear regression to the ‘Variable slope (four parameters)’ model in GraphPad Prism. For the incubation time-course studies for covalent inhibitors, the assays were carried out as above, except that M^pro^ was incubated with an inhibitor for 0, 15, or 60 min at room temperature rather than 5 min.

### Differential scanning fluorimetry

Wild-type or mutant derivatives of M^pro^ at 2.0 mg mL^−1^ in the absence or presence of 100 µM inhibitor and 40× (final concentration) of SYPRO Orange in 10 mM Tris-HCl, pH 8.0, 150 mM NaCl, 1 mM DTT, and 1.0% DMSO were heated from 20 to 95°C at a constant rate of 1°C/min on a Bio-Rad CFX96 thermal cycler. The sample volume was 40 µL. Fluorescence intensity was measured with the excitation and detection wavelengths of 450–490 and 560–580 nm, respectively. The melting temperature (*T*_m_) was determined from the peak of the first derivative of the melt curve (inflection point of the melt curve).

## Data Availability

The atomic coordinates and structure factors have been deposited in the RCSB Protein Data Bank with accession codes 9CJO, 9CJP, 9CJQ, 9CJR, 9CJS, 9CJT, 9CJU, and 9CJV.

## SUPPLEMENTAL MATERIAL

Supplemental Figures mbio.02624-24-s0001.pdf Fig. S1 to S3.

ASM does not own the copyrights to Supplemental Material that may be linked to, or accessed through, an article. The authors have granted ASM a non-exclusive, world-wide license to publish the Supplemental Material files. Please contact the corresponding author directly for reuse.
