# Supplementary material for: Structural basis for varying drug resistance of SARS-CoV-2 Mpro E166 variants
Source: mBio. 2025 Jun 2;16(7):e02624-24. doi: 10.1128/mbio.02624-24 (PMC12239574; doi:10.1128/mbio.02624-24)
Supplement: Supplemental Figures — Fig. S1 to S3. [file mbio.02624-24-s0001.pdf]

Supplemental Data

# Structural basis for varying drug resistance of SARS-CoV-2 $M^{pro}$ E166 variants

Morgan A. Esler[1,2], Ke Shi[1,2], Joseph A. Rollie[1,2], Renee Delgado[3], Jyoti Vishwakarma[3], Agnieszka Dabrowska[3], Janani Prahlad[1,2], Seyed Arad Moghadasi[1,#], Reuben S. Harris[3,4], Hideki Aihara[1,2,*]

[1] Department of Biochemistry, Molecular Biology and Biophysics, University of Minnesota, Minneapolis, Minnesota, USA, 55455

[2] Institute for Molecular Virology, University of Minnesota, Minneapolis, Minnesota, USA, 55455

[3] Department of Biochemistry and Structural Biology, University of Texas Health San Antonio, San Antonio, Texas, USA, 78229

[4] Howard Hughes Medical Institute, University of Texas Health San Antonio, San Antonio, Texas, USA, 78229

[#] Present address: NYU School of Medicine, New York, NY 10016

* Correspondence to: aihar001@umn.edu

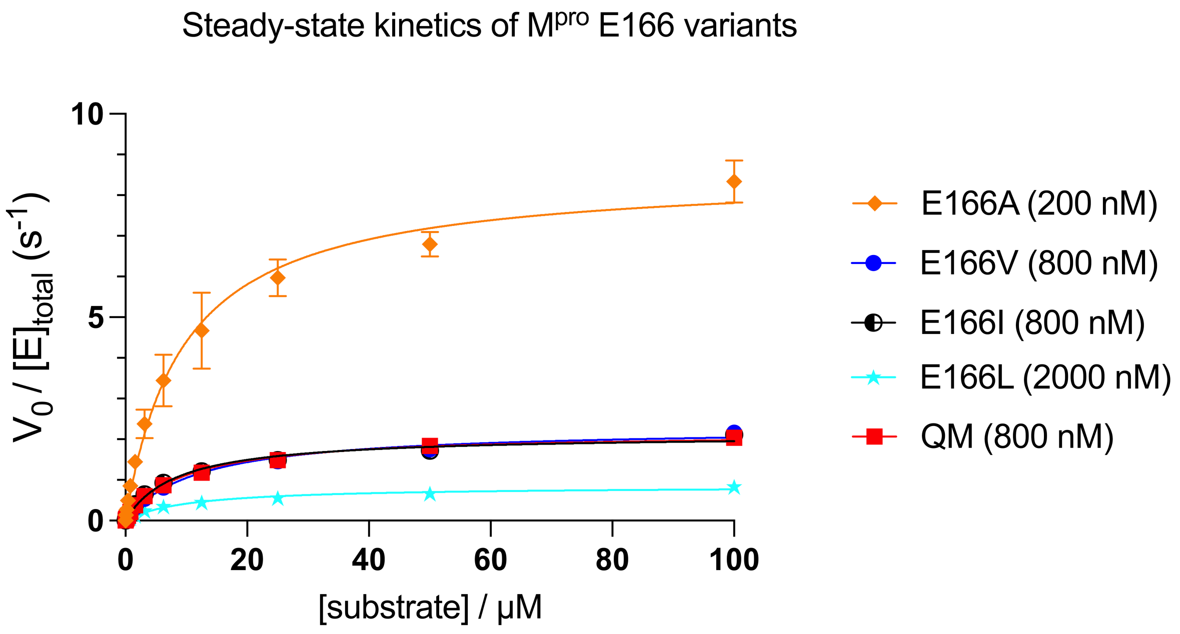


**Supplemental Fig. S1.** Michaelis–Menten plot for the $M^{pro}$ E166A/V/I/L and QM variants from Fig. 1.

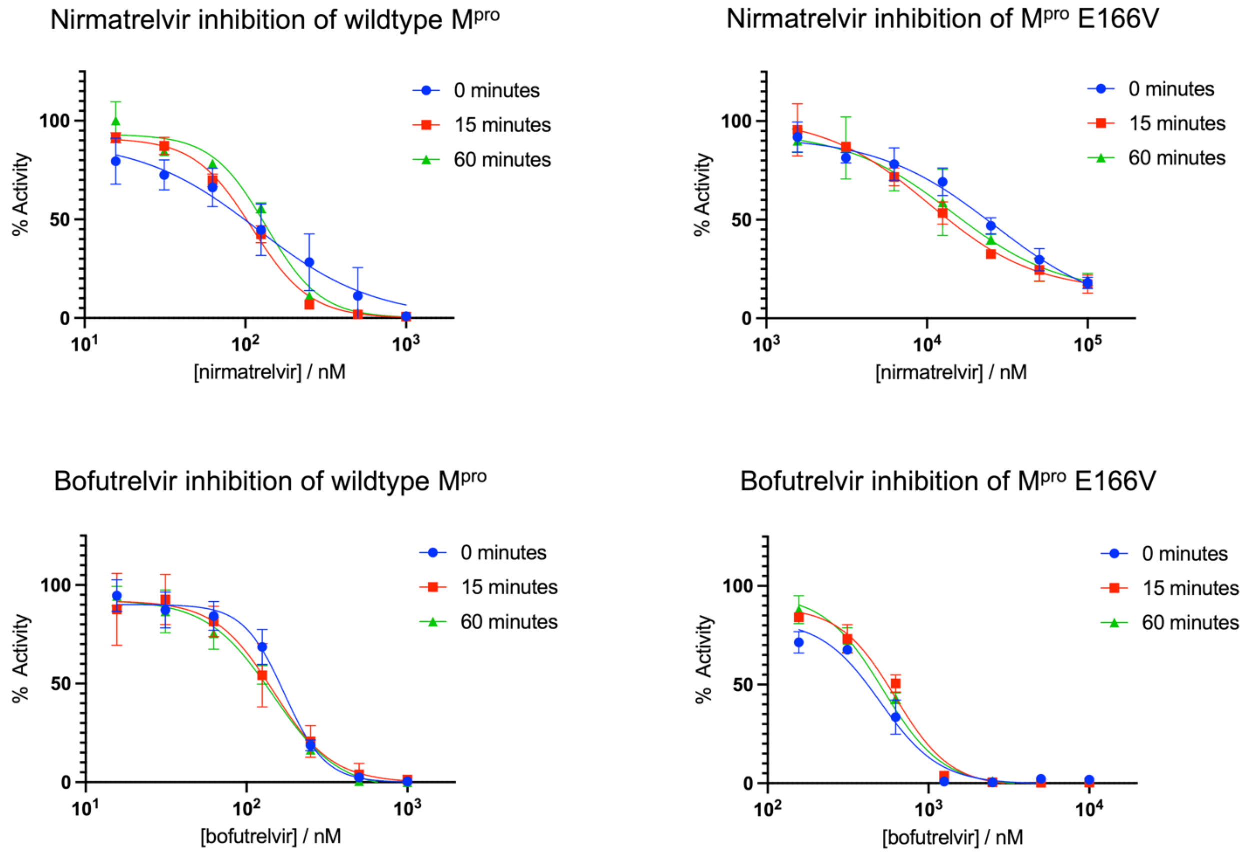


**Supplemental Fig. S2.** Dose-dependent inhibition of 200 nM wildtype or 1.0 μM E166V $M^{pro}$ by the covalent inhibitors nirmatrelvir and bofutrelvir with different enzyme/inhibitor pre-incubation times. The average values of at least 3 replicates with standard deviation are plotted.

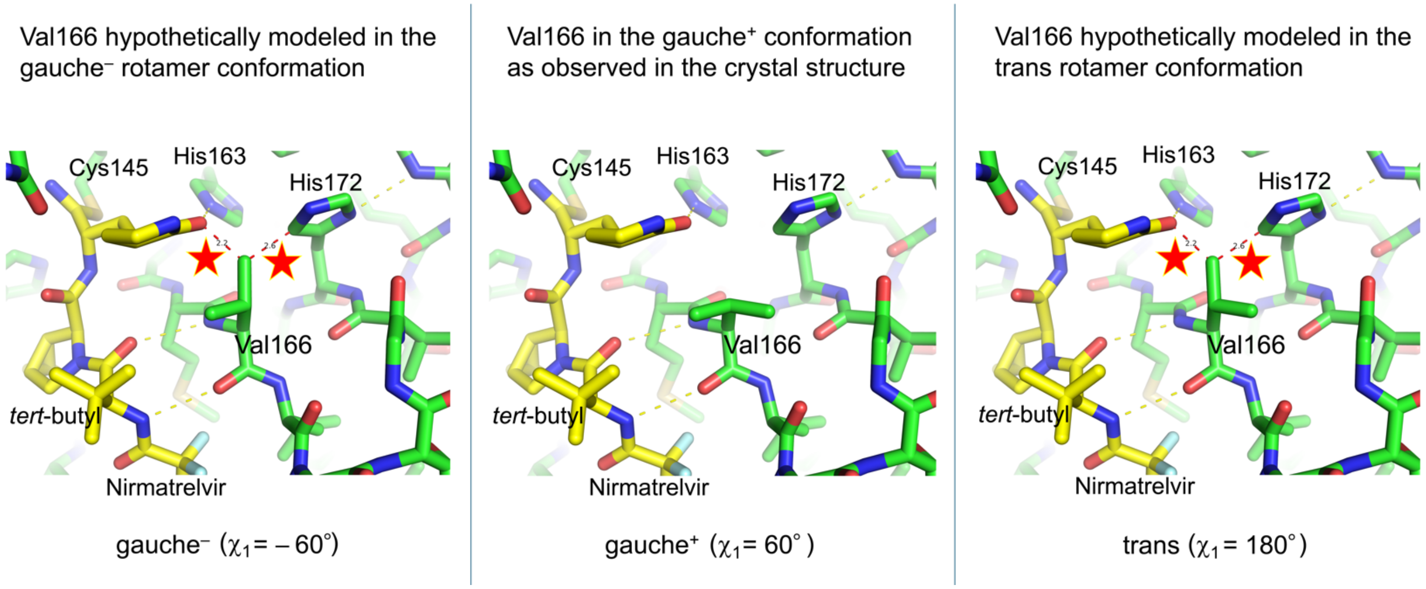


**Supplemental Fig. S3.** The side chain rotamer conformation of Val166 in $M^{pro}$ E166V is restricted to gauche$^{+}$ (middle), because either gauche$^{-}$ (left) or trans (right) would cause a steric clash with the His172 side chain as indicated by red stars. The gauche$^{-}$ or trans conformer would also severely interfere with the pyrrolidone moiety of nirmatrelvir. The only viable conformation of Val166 (gauche$^{+}$) however clashes with the *tert*-butyl group of nirmatrelvir (**Fig. 3**), leading to strong resistance. The same mechanism applies to isoleucine (E166I) with two $C\gamma$ atoms, but not to leucine (E166L) that has only one $C\gamma$ atom or alanine (E166A) without a $C\gamma$ atom.
